# Ending vaccine hegemony: Rethinking foreign aid in global health responses

**DOI:** 10.1371/journal.pgph.0004177

**Published:** 2025-01-16

**Authors:** Shreyas Teegala, Simar S. Bajaj, Oyewale Tomori

**Affiliations:** 1 Vanderbilt University, Nashville, Tennessee, United States of America; 2 Stanford University School of Medicine, Palo Alto, California, United States of America; 3 African Centre of Excellence for Genomics of Infectious Diseases, Redeemer’s University, Ede, Nigeria; PLOS: Public Library of Science, UNITED STATES OF AMERICA

Low-income countries (LICs) contend with disproportionately high rates of infectious disease mortality compared to wealthier nations [1]. Lacking the resources and financial stability to address such crises independently, they often rely on foreign aid to close critical gaps in emergency response capabilities. The COVID-19 pandemic revealed the systemic vulnerabilities of LICs during global health emergencies, highlighting both their insufficient access to vaccines and limited fiscal capacity to protect people from economic fallout [2]. International support was therefore indispensable, providing relief to marginalized nations, their institutions, and populace. While failing to offer funding support and vaccine donations, high-income countries (HICs) resorted to vaccine hoarding and did little to back the call from Global South countries for a TRIPS intellectual property waiver [3]. Many HICs, lobbied by pharmaceutical companies, also failed to support the equity and mandatory sharing clauses within the pandemic accord.

To respond to the current mpox emergency, African nations will need millions of vaccine donations, due to a shortage of trained personnel and specialized vaccine development and manufacturing infrastructure [4]. The Africa Centres for Disease Control and Prevention estimates that 10 million vaccine doses are needed to control this outbreak— a target achievable only if wealthy nations significantly increase contributions past their presently slow and inadequate pace [5,6]. This dependency, however, is part of a troubling trend in global health [7]. Although ostensibly a reasonable response to outbreaks, vaccine donations are often politically motivated, serving as subtle instruments of leverage and neocolonialism.

Vaccine donations are often justified with the moral rectitude of philanthropy and global health equity, correcting for shortages that inevitably follow infectious disease epidemics. These disparities in access have characterized several modern health crises. When concerns about swine flu broke out in 2009, HICs like the United Kingdom and United States preemptively secured sizeable vaccine agreements and negotiated priority access with pharmaceutical companies, which ultimately constrained the availability of vaccines to LICs [8]. These disparities were also apparent during the COVID-19 pandemic and 2022 mpox outbreak, when nations like the US, UK, Canada, China, and Russia domestically produced vaccines or directly brokered large vaccine orders, leaving LICs unable to secure supplies [9]. According to an estimate from the Center for Strategic and International Studies, G7 and European Union countries purchased between one and two billion excess doses of COVID-19 vaccines, which could have alleviated vaccine shortages in LICs [10]. Instead, HICs delayed and donated only paltry sums of excess vaccines in a process optimistically known as “vaccine diplomacy.”

National interests, in turn, dictate which countries receive these vaccines. For example, Russia, India, and China focused their vaccine donations on neighboring countries and key geopolitical partners, choosing not to share any doses with COVAX, a global vaccine-sharing hub that provided a mechanism for equitable vaccine allocation. Myanmar and Bangladesh were among the top recipients of vaccines from both China and India, while Russia directed its donations primarily to Belarus, Syria, and Kyrgyzstan—nations known to align with its strategic interests. Similarly, countries like Australia and several EU member states, including Poland, Romania, Hungary, and Latvia, chose to form bilateral agreements with specific states over contributions to COVAX [11]. These donation patterns highlight a preference for strategic diplomatic control over equitable allocation, revealing that national self-interests often outweighed necessity.

Take, for example, the Chinese government’s COVID-19 vaccine donations to Central and South American countries. Nicaragua received 200,000 COVID-19 vaccines a week after shifting diplomatic relations from Taiwan to China, revealing the political strings tied to such aid [12]. With the current mpox global emergency, we may see wealthier countries similarly exploit LICs’ situation to advance their own interests. In such acute crises, time wasted translates to lives lost, forcing LICs to negotiate under duress and diminishing their already limited bargaining power. Thus, they are left in a predicament to “ally or die.”

Beyond such overt, donation-based exploitation, the vaccine market serves as a new frontier for hegemony. Armed with syringes, the U.S. and China have waged a diplomatic battle for influence in Latin America and the Caribbean through COVID-19 vaccine deals. For instance, the United States structured bilateral deals with Mexico and other nearby countries, securing substantial vaccine purchases alongside donations [12]. China responded to the high volume of US vaccine shipments by offering attractive vaccine deals at lower prices, as well as $1 billion in financing for future vaccine purchases, to improve their regional influence. They strategically prioritized optics in vaccine delivery, using media-grabbing moves like chartering NFL planes to overshadow U.S. efforts [12]. Under the guise of multilateral health cooperation, HICs are leveraging vaccine deals in a neocolonial fight to consolidate influence and favor.

To replace transactional donation arrangements, we propose that the global health community restructure vaccine delivery in two ways. First, HICs should reallocate the bulk of their aid from vaccine donations to foreign direct investment (FDI) in LICs’ vaccine infrastructure. This approach shifts aid dollars from reactive crisis management to proactive capacity building, laying the foundation for future public health responses. FDI presents its own set of challenges, so vaccine investments must be thoughtfully structured. For example, the arrival of foreign business can disrupt existing market conditions, but by forming joint ventures with local industry, HICs and multinational corporations can mitigate competition with local businesses and foster mutually beneficial relationships. Insufficient demand can also undermine these investment efforts, rendering them financially unsustainable. Take Moderna’s April decision to cancel its plans for an mRNA vaccine manufacturing facility in Kenya, for example. Initially hailed as a major step toward addressing vaccine inequities by building local production capacity, the project was ultimately abandoned due to projected shortfalls in vaccine demand [13]. To this end, the global health community could subsidize vaccine purchases between African nations, in lieu of donating doses, to help maintain demand and mature local enterprises. Supporting programs like the African Vaccine Manufacturing Accelerator, which is committed to purchasing 800 million vaccines from Africa in the next decade, could help ameliorate these concerns as well.

The second strategy regards international stakeholders increasingly supporting South-South collaborations to strengthen vaccine development and manufacturing in the Global South. The hub-and-spoke model has previously helped equitize COVID-19 vaccine distribution by empowering African scientific capacity [14], and it may guide this paradigm shift as well. With the current crisis, a central hub, such as an mRNA vaccine facility, could rapidly develop new mpox vaccine technology (as well as future vaccine developments) and distribute it to various regional spokes across Africa. This hub could be established with funding from the WHO, or perhaps FDI, while the spokes generate ongoing demand in support. African nations can thus develop sustainable domestic infrastructure for future outbreaks, reducing their reliance on foreign aid and vulnerability to hegemonic interests. Indeed, this is the vision for Africa Union, which has articulated “A New Public Health Order for Africa”, which aims to strengthen the self-sufficiency of African public health, including regional manufacturing of vaccines, medicines and other tools [15].

To effectively address the manifold health emergencies the world will inevitably face, mutual collaboration must replace opportunistic philanthropy with sustained capacity building. The best way to fight rampant pathogens that know no borders is by building systems that transcend them.
